# The Demoiselle of X-Inactivation: 50 Years Old and As Trendy and Mesmerising As Ever

**DOI:** 10.1371/journal.pgen.1002212

**Published:** 2011-07-21

**Authors:** Céline Morey, Philip Avner

**Affiliations:** Institut Pasteur, Unité de Génétique Moléculaire Murine, CNRS, URA2578, Paris, France; Medical Research Council Human Genetics Unit, United Kingdom

## Abstract

In humans, sexual dimorphism is associated with the presence of two X chromosomes in the female, whereas males possess only one X and a small and largely degenerate Y chromosome. How do men cope with having only a single X chromosome given that virtually all other chromosomal monosomies are lethal? Ironically, or even typically many might say, women and more generally female mammals contribute most to the job by shutting down one of their two X chromosomes at random. This phenomenon, called X-inactivation, was originally described some 50 years ago by Mary Lyon and has captivated an increasing number of scientists ever since. The fascination arose in part from the realisation that the inactive X corresponded to a dense heterochromatin mass called the “Barr body” whose number varied with the number of Xs within the nucleus and from the many intellectual questions that this raised: How does the cell count the X chromosomes in the nucleus and inactivate all Xs except one? What kind of molecular mechanisms are able to trigger such a profound, chromosome-wide metamorphosis? When is X-inactivation initiated? How is it transmitted to daughter cells and how is it reset during gametogenesis? This review retraces some of the crucial findings, which have led to our current understanding of a biological process that was initially considered as an exception completely distinct from conventional regulatory systems but is now viewed as a paradigm “par excellence” for epigenetic regulation.

## A History of X-Inactivation: Early Studies (1950–1980)

**Figure 1:**
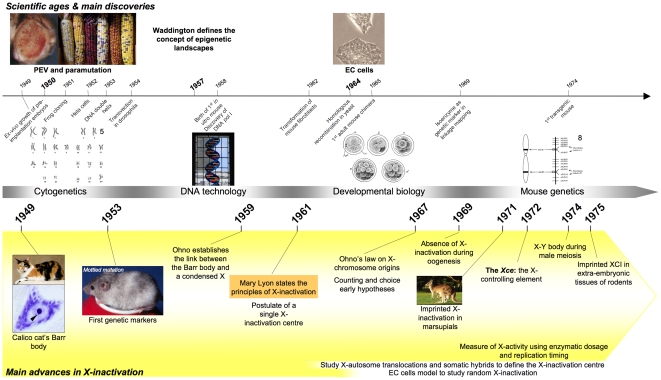
Timeline showing milestones in the history of X-inactivation (1950–1975). Images are taken from http://commons.wikimedia.org, are a courtesy of the corresponding authors, or are unpublished data.

The 1950s and the decades that followed provided much of the basis for present-day developmental biology and molecular genetics (Figure 1). It was a period of crucial advances in mammalian embryology (e.g., *ex vivo* growth of mouse embryos [1], [2] and transgenic experiments [3]). Contemporary description of the DNA double-helix [4], of homologous recombination [5], of cloning [6], and of the first DNA-based genetic markers [7] similarly opened up the path for genetic engineering, extensive genetic mapping, and seemingly extraordinary quirky observations such as those concerning Position Effect Variation (PEV) in *Drosophila* [8], [9]. McClintock's earlier work on transposable elements in maize [10] could, moreover, increasingly be assimilated and interpreted with reference to the intellectual context provided by work such as Jacob and Monod's on the genetic regulation of the *lac* operon [11]. The new and seemingly quirky kinds of gene regulation that could not be explained by Mendelian genetics *per se* laid the groundwork for the concept of epigenetics—a term derived from the fusion of “genetics”, referring to the primary DNA code, and “epigenesis”, referring to the differential interpretation of the hereditary material within different cell lineages—as being, at least in part, responsible for the relationship between genes and phenotypes [12].

The conditions and nature of the discovery of X-inactivation in the early 1960s illustrate perfectly both the intellectual burgeoning that characterised these years and the emergence of the concept of epigenetics.

### The Discovery of X-Inactivation

In 1949, the scrutiny of motoneurons of a female calico cat by Barr and his PhD student Bertram led to the identification of a dark, condensed structure situated close to the nucleolus [13]. Whilst Barr and Bertram did not realise at the time that they were looking at an inactive X chromosome (Xi)—the critical link between the “Barr” body and a condensed X chromosome was to be made only later by Susumu Ohno [14], [15]—their observation, along with that relating to the description of two X-linked loci, *Tabby* and *Mottled*, able to confer a mosaic coat colour to heterozygous females [16], and the realisation in 1959 that XO female mice were able both to develop normally and to reproduce [17], were critical to the formulation by Mary Lyon of the X-inactivation theory (for early reviews relating to the discovery of X-inactivation, see [18]–[20]).

In her key 1961 publication, Mary Lyon suggested that the heterochromatic X could correspond in different somatic cells of the same female mammal either to the maternally inherited or to the paternally inherited X chromosome, and proposed that a process leading to the global silencing of the genes of an entire X chromosome referred to as “X-inactivation” occurred during early embryogenesis and was clonally inherited thereafter, thus providing an explanation for the tortoiseshell pattern of Barr's calico cat [21]. Similar ideas were also advanced by Beutler and colleagues to account for their observation of the presence of two types of red cell in human females heterozygous for the X-linked deficiency in glucose-6-phosphate dehydrogenase (*G6pdx* gene) [22] and by Russell, who put forward a similar—if less elaborate—explanation for variegation in female mice carrying X-autosome translocations [23].

### Counting, Choosing, and Skewing

Mary Lyon's theory prompted researchers to study individuals carrying more than one X per set of autosomes. Surprisingly, independently of the configuration, all but one of the X chromosomes in the cell were observed to be condensed, suggesting that each cell could “count” the number of X chromosomes and accordingly inactivate (n−1) Xs per autosome set [20]. This presumed counting process would therefore be responsible for the absence of X-inactivation in male cells.

Other surprising observations concerned the concept of “choice” of active and inactive X(s) and the molecular mechanisms ensuring randomness. Non-randomness, or skewing, can be caused by secondary selection for or against cells carrying the active or the inactive X chromosome (for review see [24]) or alternatively by primary non-random choice occurring during the X-inactivation process itself. The latter implies that a distortion from the 1∶1 ratio of X-inactivation in diploid cells can be caused by factors/genomic region(s) implicated in the X-inactivation process itself. An example of primary skewing is the X-controlling element (*Xce*), a mouse locus defined in 1972 by Bruce Cattanach, after crosses of mice on different genetic backgrounds revealed that some Xs were more likely to resist X-inactivation than others depending on the *Xce* allele they carried [25]. No locus homologous to *Xce* has as yet been described in the human, possibly due to the difficulties of conducting similar analyses.

### Developmental Regulation of X-Inactivation

Another key issue at this time was the establishment of where and when X-inactivation took place during development. In the mouse, the Xs that originate either from spermatogenesis, where the paternal X is sequestered within the “sex body” (for review see [26]), or from the female germline, where the maternal X undergoes reactivation at the onset of meiosis, were both shown to be active in the fertilised egg and to remain active until the 8-cell stage as measured by biochemical studies of the few available X-linked isoenzymes [27], [28]. Such early biallelic expression was suspected to concern only a few genes and/or to be of low level and therefore tolerated at these early embryonic stages. The first wave of X-inactivation was originally thought to occur around E3.5 in the extra-embryonic tissues of the trophectoderm and of the primitive endoderm and to consist in a preferential inactivation of the paternal X (imprinted X-inactivation) [29]. In contrast, random X-inactivation was identified as occurring around the time of implantation (E5.5) in cells of the epiblast that give rise to the embryo proper [30], [31]. Of note, the description of imprinting as part of the X-inactivation anticipated by several years the first reports of parental imprinting at autosomal loci [32], [33].

These early studies resulted in X-inactivation being firmly established as the major mechanism responsible for dosage compensation of X-linked gene expression between the sexes in mammals, with the characterisation of a small number of key characteristics such as late replication timing and condensed heterochromatic structure allowing the Xi to be reproducibly distinguished from its active homologue.

## The X-Inactivation Centre and the *Xist/XIST* Gene (1970–2000)

**Figure 2:**
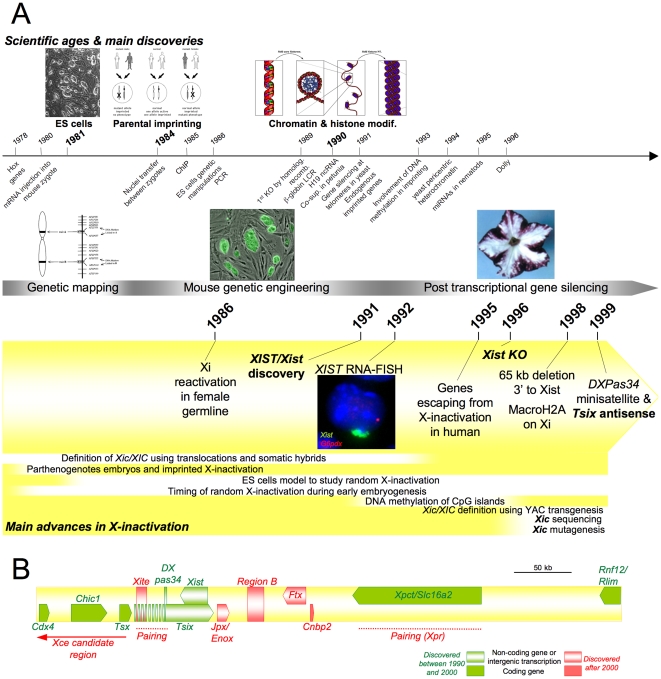
Main discoveries of the years 1975 to 2000. (A) Timeline showing milestones in the history of X-inactivation (1975–2000). Images are taken from http://commons.wikimedia.org, are a courtesy of the corresponding authors, or are unpublished data. (B) Map of the mouse *Xic*.

Intuitively, both counting and choice had to require elaborate mechanisms of a new kind involving both the *trans* communication between Xs and between X chromosomes and autosomes and the *cis* propagation of the X-inactivation signal along the entire chromosome. Both functions were postulated to be controlled by a single X-linked region called the X-Inactivation Centre (*Xic/XIC* in mouse/human) from which the X-inactivation signal would then spread to the rest of the chromosome [34]. Retrospectively, it appears relatively visionary to have imagined such a region capable of chromosome-wide concerted gene silencing, especially considering that long-range *cis*-regulations such as the β-globin Locus Control Region were reported only considerably later [35], [36]. Paradoxically, the *trans* effect, which now seems particularly intriguing, may have appeared, at the time, as something relatively common given the fact that transvection in *Drosophila* had been described by Ed Lewis some 29 years earlier [37] (Figure 2; for review, see [38]).

### Defining the X-Inactivation Centre (*Xic/XIC*) Using Chromosome Rearrangements and Transgenesis

The hunt for the *Xic/XIC* was initially engaged in the human by comparing a battery of X-autosome translocations that had been identified in clinical research centres. Translocation breakpoints were determined cytologically using chromosome banding patterns and X-inactivation profiles were assessed through replication timing. These experiments resulted in the human *XIC* candidate region being restricted to an interval of some 660–1,200 kb [39]. Similar approaches led to a much larger genetic interval of 8 CM being defined in the mouse [40], [41]. Importantly, both series of studies confirmed the original hypothesis that a single X-linked region—and not several interspersed loci—underlay *Xic/XIC* function. Other experiments using mouse translocations showed that inactivation was able to spread from the Xi into attached autosomal material, indicating that the propagation of X-inactivation probably involved mechanisms similar to PEV in *Drosophila* rather than mechanisms depending exclusively on X-specific sequences [42].

Early observations on female Embryonal Carcinoma (EC) cells [43] that had suggested that such cell lines might prove useful for X-inactivation studies [44] were confirmed and amplified by the derivation of male and female Embryonic Stem (ES) cells, which were shown to recapitulate, upon *ex vivo* differentiation, the steps leading to stable random X-inactivation. The concomitant development of large fragment transgenesis using these ES cells and embryos permitted the pursuit of *Xic/XIC* function using Yeast Artificial Chromosomes (YACs) first, then P1 phages and cosmids carrying different *Xic* formats [45]–[48]. These studies allowed the minimal *Xic* region necessary for both random X-inactivation and imprinted X-inactivation to be defined [45], [49]. An experimental rider to the 450-kb region defined as necessary for random X-inactivation is the multicopy nature of the transgene array used [50] (for review see [51]).

### The *Xist/XIST* Non-Coding Gene

The search for an *XIC* candidate gene led to the isolation of the *XIST* gene based on its specific expression from the human Xi (hence its name, X-inactive specific transcripts) [52]. Though the human and mouse *Xist* homologues are relatively poorly conserved at the sequence level, both lie within the *XIC*/*Xic* and show similar overall genomic organisation [53]–[56]. Both *XIST*/*Xist* genes produced very large transcripts (15–17 kb) restricted to the nucleus that do not code for a protein. In this respect, *Xist/XIST* constituted one of the first large non-coding RNAs to be discovered, not long after the *H19* RNA involved in the regulation of the imprinted locus *Igf2/H19* was described [57].

The need to follow the behaviour of the inactive and active X chromosomes within the context of a single nucleus led to the rapid implementation of single cell analyses such as fluorescence in situ hybridisation (FISH) techniques. This allowed the visualisation of *XIST* RNAs within female somatic nuclei as an accumulation or decoration of the Xi, suggesting a possible structural role for the *Xist/XIST* transcripts [54], [58]. Additionally, kinetics of *Xist* expression during early mouse development revealed that *Xist* was expressed as early as the 4-cell stage from the paternal X, suggesting early onset of imprinted X-inactivation in the embryo [59], [60]. The lack of inactivation of an X chromosome mutated for *Xist* confirmed the major role of the gene in X-inactivation initiation [61], [62].

### *Xist/XIST* Does Not Resume All *Xic/XIC* Functions

During this period, major positional cloning efforts using genetic and physical mapping resulted in the first large-scale sequencing of *Xic* subregions [63]. Several new genes and putative functional elements within the *Xic/XIC* interval were identified. Amongst them, the *DXPas34* minisatellite lying 16 kb downstream of *Xist* appeared to share significant properties with imprinting centres governing the monoallelic expression of autosomal imprinted clusters such as differential DNA methylation profiles [64] and associated long-range non-coding transcription running antisense to *Xist* [65]. The *Xce* locus was also shown to map to the *Xic* region and to be distinct from *Xist* [66], although its precise location [67], nature, and action remain undetermined.

The establishment of *Xic* physical maps and genomic sequencing also provided the tools to generate targeted mutations of specific *Xic* elements and regions. Such mutagenesis notably allowed the creation of a large deletion encompassing 65 kb of sequence 3′ to *Xist*, which resulted in a systematic inactivation of the mutated X regardless of the presence of another X chromosome in the cell [68]. At the time, this striking phenotype was interpreted as identifying a counting element within the deleted span, thereby irrevocably showing that *Xist* did not recapitulate all *Xic* functions.

## Main Discoveries since the Year 2000 and Pending Questions (2000–Present)

**Figure 3:**
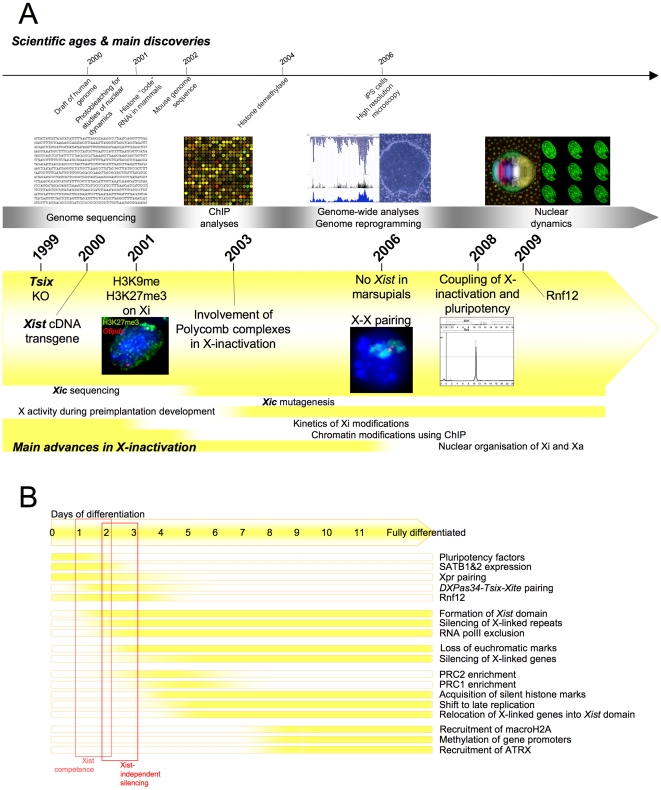
Main discoveries of the years 2000 to 2011. (A) Timeline showing milestones in the history of X-inactivation (2000–2011). Images are taken from http://commons.wikimedia.org, are a courtesy of the corresponding authors, or are unpublished data. (B) Kinetics of events leading to fully stable inactive state during the differentiation of female mouse ES cells.

During the new millennium, progress in gene targeting facilitated the creation of a large variety of novel mutations within the *Xic* that have considerably improved our understanding of X-inactivation initiation. In parallel, the emergence of a role for chromatin structures as putative transcription regulators [69], [70] and the development of Chromatin Immuno-Precipitation (ChIP) techniques allowing analysis of chromatin composition [71] has strongly impacted our ideas of the mechanisms involved in X-inactivation, building in this respect on earlier documented changes in Xi-associated global histone hypoacetylation [72] and CpG island methylation [73], [74]. These experiments have underlined the likely integrated multi-level and redundant nature of the mechanisms ensuring the stability of the inactive state. Additionally, the finding that lineage specific genome programmes could be efficiently reverted to the pluripotency state(s) as demonstrated, notably, by female induced Pluripotent Stem (iPS) cells [75] and that this was accompanied by Xi reactivation [76] has reinforced interest in the link(s) between cell differentiation and X-inactivation triggering suggested by ES cell differentiation studies. Finally, the many studies of gene nuclear organisation that have shown that chromatin fibres do not fold randomly but rather in a dynamic and directed manner that is correlated with gene expression status [77] have strongly encouraged the investigation of these topological and dynamic aspects of X-inactivation (Figure 3).

### *Tsix* and the Transcription Antisense to *Xist*

In the mouse, the enigma of the transcription antisense to *Xist* was resolved with the description of *Tsix*, a non-coding gene whose major promoter is located just upstream of the *DXPas34* minisatellite [78]. Interestingly, *Tsix* function does not seem to be conserved in other species (see below). The targeted deletion of *Tsix* [79]–[81] or of *DXPas34* [82], [83] induced a drastic reduction of *Tsix* transcription that resulted in the preferential inactivation of the mutated X in differentiated female cells. This indicated that *Tsix*/*DXPas34* is involved in the repression of *Xist* in pluripotent ES cells and in random choice during differentiation [84], [85]. The implication of *Tsix* in imprinted X-inactivation has also been inferred from the absence of apparent effect of paternally inherited *Tsix* mutations as opposed to ectopic *Xist* expression and embryonic lethality associated with maternal transmission [80], [86]. The role of *Tsix* in the counting process has been addressed by targeting *Tsix* mutations to XO or XY cells. In the majority of cases such mutations result in ectopic X-inactivation, thereby pointing to a role of *Tsix* in the counting process [68], [81], [82], [85], [87], although one report suggests otherwise [79]. The divergence in phenotypes in these studies has been suggested to be linked to variations in the differentiation protocols under use.

The emergence of regulatory antisense RNAs has raised a series of questions as to their underlying mechanism(s) of action. Does it necessarily involve RNA interference (RNAi) [88]–[90]? Or RNApolII activity across the target genes? Or the induction of local chromatin modifications? The investigation of these issues has implicated *Tsix* transcription in maintaining an open chromatin structure along the *Xist* gene [91]–[93] and in the setting up of a specific chromatin configuration at the *Xist* promoter [94]. This activity does not appear to be critically dependent in *Tsix* splicing [95]. Despite extensive community efforts, no conclusive evidence for a role of siRNAs involving the *Xist*/*Tsix* overlap has been adduced and the single report of such activity has yet to be confirmed [96]. The absence of an RNAi-based mechanism as the main mediator of *Xist* repression is in agreement with the absence of a drastic X-inactivation phenotype in ES cells mutated for an essential member of the RNAi machinery, Dicer [97], [98].

### In-Depth Characterisation of *Xist* Expression and the Molecular Function(s) of *Xist* RNA

The fascinating visualisation of *Xist/XIST* RNAs “decorating” the Xi in *cis* but not in *trans* in a developmentally regulated manner has prompted researchers to investigate the molecular mechanisms behind *Xist/XIST* action. Keynote insights have come from a series of experiments based on the use of inducible *Xist* cDNA transgenes in male ES cells, a system that allowed the over-expression of *Xist* at different time points during differentiation. With the possible rider that these studies involve the generation of non-physiological *Xist* expression levels and the use of *Xist* as a spliced form, a major finding was that of a critical window of time during which *Xist* was competent to induce transcriptional repression and after which the chromosome becomes refractory to silencing and the maintenance of gene repression is *Xist* independent [99]. The existence of a “chromosomal memory” suggested by the observation of more efficient initiation of X-inactivation in cells that had experienced earlier *Xist* exposure was also postulated [99].

Using mutations within the *Xist* cDNA, the silencing function was attributed to the highly conserved repeat A located at the 5′ end of the transcript, whereas the rest of the molecule appears to participate in the coating of the Xi in a synergistic, if partially redundant, manner [100]. Another repeat (repeat C) also interacts with a nuclear matrix attachment protein—hnRNP-U/SAF-A—and this interaction is necessary for correct *Xist* coating [101]. These results may explain the long-standing observation that *Xist* RNAs remained attached to the nuclear matrix after chromatin extraction [58], suggesting that *Xist* transcripts interact with the nuclear scaffold rather than directly with the Xi (for review see [102], [103]). *Xist*-mediated mechanism(s) might also involve—albeit probably indirectly—the SATB1 and SATB2 nuclear matrix attachment proteins [104]–[106].

### Chromatin Modifications, Chromatin Remodellers, and Their Role in the Establishment and Maintenance of Silencing

In the noughtie years, multiple experiments were aimed at indexing the chromatin modifications that characterise the Xi in the hope of reconstructing the chain of events leading to the fully locked inactive state. One of the strategies employed involved using immuno-fluorescence combined with *Xist* RNA-FISH at successive time points during female ES cell differentiation [107]. A sequential ordering was described with *Xist* coating of the Xi as the trigger rapidly followed by RNApolII exclusion, the loss of euchromatic marks and almost concomitantly the recruitment of the Polycomb group complex PRC2 [108]–[111], then PRC1 [112] with the consequent accumulation of the heterochromatin marks H3K27me3 and H2AK119ub. Other heterochromatic marks, histone variants such as macroH2A [113], chromatin remodellers (ATRX) [114], and CpG island methylation were other later apposed modifications (for details of the kinetics and the nature of the modifications see [115]).

The number and variety of epigenetic changes—including those still to be uncovered—highlights the extent and depth of the progressive metamorphosis that the presumptive X undergoes during X-inactivation. Although the regional organisation of these different marks along the length of the Xi remains to be established, some ChIP data have already revealed that some marks such as H3K27me3 are preferentially associated with promoters and gene bodies [116], and others, such as the macroH2A histone variants, are more globally distributed [117]. Interestingly, whilst DNA methylation was observed at Xi gene promoters—albeit quite heterogeneously—genes on the active X were hypomethylated at the promoter and hypermethylated in the body of the gene [118]. ChIP analyses on the *Xic* region have suggested that the presence of specific chromatin domains along the *Tsix/Xist* locus and upstream of *Xist* prior to the onset of differentiation is important for X-inactivation randomness [93], [119], [120], but stringent analysis of the specific function of the individual epigenetic marks is still mostly lacking.

### Revisiting the Kinetics of X-Inactivation during Pre-Implantation Development

A fundamental question regarding the nature of the imprint on X chromosomes has been to clarify whether the paternal X enters the oocyte in an already “pre-inactivated” state that is subsequently maintained, implying that paternal genes would be silent from the zygotic stage onwards. This question has been the theatre of both lively debate and extensive work. RNA-FISH analysis of several genes interspersed along the paternal X during pre-implantation have now led to the consensual view that an additional reactivation of the paternal X must occur at some point between the onset of spermiogenesis and the 2- to 4-cell embryo stage [121]–[123]. These analyses also revealed that genes on the paternal X were not silenced synchronously, suggesting that the initial repressive state involves genes or possibly region-specific mechanisms.

The evidence of *de novo* imprinted X-inactivation during pre-implantation development [111], [124], [125] favours the existence of a robust imprint acting to prevent the inactivation of the maternal X at these stages. This hypothesis is supported by previous observations on gynogenetic embryos where the absence of imprinted X-inactivation was accompanied by the death of the embryos around implantation, in contrast to androgenetic embryos, which were capable of achieving regular random X-inactivation and of surviving until E7.7 [59]. This imprint could be mediated by a strong repression of *Xist* (as illustrated by the total lack of expression from the maternal *Xist* locus compared to a pinpoint expression from the paternal locus [125]), although the requirement of *Xist* for the triggering of imprinted X-inactivation has recently been questioned [121].

### Linking X-Inactivation to Pluripotency and Genome Reprogramming

The long-searched-for link between cellular differentiation and X-inactivation was recently established through the discovery that pluripotency factors Nanog, Oct3/4, and Sox2 bind to *Xist* intron 1 to prevent *Xist* upregulation in undifferentiated ES cells [126] whilst the pluripotency factors Rex1, Klf4, and c-myc occupied the *Tsix* promoter and activated *Tsix* expression [127]. As a consequence at the onset of differentiation, the loss of these pluripotency factors would be expected to be associated with the induction of *Xist* upregulation. Whilst it is clear that additional binding sites of pluripotency factors/developmentally regulated factors within the *Xic* remain to be uncovered [128], these important results suggest a direct connection between Xi reactivation during experimentally induced pluripotency and the molecular mechanisms responsible for the genome-wide resetting occurring in the inner cell mass (ICM) (for review see [129], [130]).

It is striking that Nanog has also been detected in female Primordial Germ Cells (PGCs) from E7.75 onwards, a time when Xi reactivation has been shown to initiate [131]–[133], indicating that Nanog might also be involved in Xi reprogramming in the female germline (for review see [134]). Intriguingly, however, Xi reactivation appears to occur progressively throughout the time of PGCs' migration to the genital ridge, thereby dramatically contrasting with the speed of reactivation occurring in the ICM. This suggests that slightly different and as yet uncharacterised mechanisms may be at work during one of the types of reactivation. Another related question concerns the absence of reactivation of the paternal Xi during early pre-implantation despite the expression of some of the key pluripotency factors. An attractive working hypothesis is that parental imprinting at these stages prevents the action of the pluripotency factors. The lack of Xi reactivation in the epiblast (and in derived female EpiStem Cells [135]) raises similar issues, although at this later stage, the absence of some pluripotency factors such as Nanog and Rex1 thought to be required for the initial *Xist* repression [126] may be sufficient explanation.

### Nuclear Dynamics and *trans*-Communication between X-Chromosomes

Large-scale nuclear reorganisation has been shown to accompany the establishment of random X-inactivation. 3D-FISH analyses suggest that the core of the Xi chromosome territory is constituted of non-genic sequences, including LINE-1 repeats that provide the support for the initial coating by *Xist* RNAs [136]. This is followed by global chromatin changes and by the relocation of genes to within the *Xist* repressive compartment [137]. These observations favour another of Mary Lyon's hypotheses, who proposed, based on an enrichment of the X chromosome for LINE-1 elements, that the latter serve as “way-stations” facilitating the propagation of the inactivation signal [138], [139].

Nuclear dynamics may also be implicated in X chromosome counting and random choice. It has recently been observed that the two X chromosomes come into close nuclear proximity both before and at the very beginning of the differentiation process and that these X-X pairing events [61] involve two specific regions within the *Xic*, respectively: the *Xpr*, located within the *Xpct* gene [140], and the *DXPas34-Tsix*-*Xite* region [141], [142], which has long been suspected of participating in both counting and choice. Dynamic nuclear contacts between these regions are thought to mediate the *trans*-sensing of the two X chromosomes and to resolve through the apposition of distinct modifications on each allele, resulting in transient asymmetric *Tsix* expression [143]. This would then provide a window of opportunity for monoallelic *Xist* upregulation (for a review on nuclear organisation during X-inactivation, see [144]).

## Changing Our Attitudes: The Evolution of X-Inactivation Mechanisms

X-inactivation in “ancient mammals” such as the marsupial is characterised by unstable imprinted inactivation of the paternal X, and, on this basis, imprinted X-inactivation was hypothesised until the mid-1990s to represent the ancestral form of X-inactivation [145]. This form of X-inactivation was thought to have been partly conserved in the mouse, which displays imprinted X-inactivation both during pre-implantation development, prior to the onset of random X-inactivation [111], [124], [146], and in extra-embryonic tissues [29], whereas hominids appear to have evolved towards the complete replacement of imprinted by random X-inactivation [147], [148] (reviewed in [149]). Crucial insights into our understanding of the evolution of X-inactivation mechanisms have come from recent sequence comparison of the X-inactivation centres of different species [150], [151]. These showed that *Xist/XIST* has evolved from a protein coding gene present in marsupials, indicating that other non-coding RNAs or totally different mechanisms must be at work in such “ancient mammals” [152]. *Xic/XIC* sequence comparisons had previously shown that the human *TSIX* was either completely absent or present in a truncated form, resulting in an absence of antisense transcription at the *XIST* promoter [150], [153], [154] (for review see also [155]). In parallel, other studies have led to the identification of several new non-coding genes (*Jpx/Enox* and *Ftx*) in the *Xic*, showing various degree of conservation [150]. Taken together, these analyses underline the surprising evolutionary instability of the master region controlling X-inactivation and of some of the key actors identified as critical in functional studies in the mouse.

Other important mechanistic differences have been identified through transgenic experiments. For instance, a YAC transgene containing the entire human *XIST* when integrated into the mouse genome, unlike the endogenous mouse *Xist* gene, initiated X-inactivation even before differentiation [156], [157]. This points to a conservation—totally or partially—of the mechanisms involved in the *cis*-spreading of X-inactivation between the two species together with a lack of conservation of the mechanisms acting to ensure *XIST cis*-repression prior to differentiation. The latter may be associated with the absence of human *TSIX* (see above). Interestingly, a recent comparison of X-inactivation profiles during pre-implantation development in humans and rabbits has found a late onset of X-inactivation in both species compared to mice and initial biallelic upregulation of *Xist* alleles prior to monoallelic resolution [158]. Additional species-specific differences include the recruitment of diverse heterochromatin marks in marsupials, mice, and humans [159]–[162].

A last but certainly not least difference between mice and human concerns X-linked genes escaping from X-inactivation. In humans, unlike mice [163], a large number (15%) of X-linked genes have been shown to escape from X-inactivation [164], offering a potential explanation of the severity of the phenotypic alterations observed in XO women (Turner Syndrome) compared to mice (for review see [165], [166]). A level of variability in the degree of escape has also been reported between individuals, between tissues, and even amongst cells of the same tissue. Interestingly, the distribution of the genes escaping from X-inactivation along the chromosome also differs between human and mouse. In mice, the few “escapees” are either embedded within regions undergoing X-inactivation or located within the single murine Pseudo-Autosomal Region (PAR) (shared with the Y chromosome). In humans, genes escaping from X-inactivation are similarly found in both human PARs but, in addition, exist within clusters in large genomic domains that may be several megabases in size. This suggests that large-scale chromatin remodelling as opposed to gene-based mechanisms is likely at work in humans [163], [164], [167]. In mice, LINE-1 transcription [136], the expression of other non-coding RNAs [168], and binding of the insulator CTCF [169] at the boundaries of escapees are associated with the looping out from the *Xist*-repressive compartment [137], which is thought to participate in preventing the spreading of heterochromatin into genes that escape from X-inactivation. Transgenesis approaches allowing the introduction of escapees into different genomic contexts should enable the further dissection of the molecular mechanisms underlying this phenomenon [170].

An unexpectedly large variety of mechanisms involved in the initiation, spreading, and stabilisation of X-inactivation therefore probably exist in the mammalian kingdom. This suggests that “a la carte” mechanisms most likely evolved to adapt to, and cope with, the developmental and gestational specificities of each species. The original observation of the dense Barr body led researchers to postulate a chromosome-wide process that would affect the entire X chromosome uniformly. The more recent findings suggest that gene- or gene cluster-based mechanisms allow the fine tuning of X-inactivation to cope with the specific requirements of development and/or tissue/lineage functionalities. Such mechanisms may be related to systems used in other phyla to compensate sex chromosome dosage, as in birds, where only few genes are subject to dosage compensation [171], [172], or in *Drosophila*, where X over-expression in males is initially established preferentially and locally at entry sites scattered all along the X [173].

## Concluding Remarks

As the inactivation traveller looks back over the 50 years since Mary Lyon's original hypothesis was published, it seems that quite a long—if winding—road has been covered and some great achievements made. Raising our eyes, however, reveals the extent of the path still in front of us.

Moreover, earlier X-inactivation travellers, like Himalayan climbers, have left their load of unresolved issues. For instance, despite intense scrutiny and in-depth mutagenesis studies, we still mostly ignore how the *XIC*/*Xic* exerts its function, and even *Xist*'s mode of action remains rather obscure. A role for *Xist* in recruiting the chromatin remodeller PRC2 [174], which, in turn, triggers H3K27 trimethylation, has found support from similar results obtained with other large non-coding RNAs such as *Air/AIR*, *Kcnq1ot1/KCNQ1OT1* (regulation of imprinted genes at the *Igf2r/IGF2R* and at the *Cdkn1c/CDKN1C* loci), and *HOTAIR* (developmental regulation of *HOXD* gene cluster in human) [175], [176]. The recent observation that the mutation of the mouse *Hotair* was without dramatic impact on the regulation of the mouse *Hoxd* cluster [177] provides a welcome cautionary reminder of the need to cross-reference such studies to *in vivo* functional approaches. We also still ignore how the original euchromatic marks are removed from the Xi. Does this require the association of *Xist* RNAs with specific histone demethylases, or does it depend solely on the passive dilution occurring via DNA replication and/or successive mitoses? Other *Xist/XIST*-related questions concern the potential role of *Xist/XIST* splice variants—are they just relics of evolution? Or integral to the resetting of the *Xist/XIST* domain after DNA replication or mitosis?

Within the *Xic*, the function of many of the more recently discovered non-coding RNAs such as *Jpx/Enox* [178], [179] and *Ftx* [180] and of sites of intergenic transcription such as *Xite* [181] and the *Region B* [150] remains to be fully elucidated, as does the role of actors lying outside of the immediate *Xic/XIC* interval, which are involved in the counting process. The U3 ubiquitin ligase produced by the X-linked *Rnf12* gene, which was recently shown to act on the initiation of X-inactivation in a dose-dependent manner, is the first of such actors to be characterised [182]–[184].

The concentration of research into understanding how the *Xic/XIC* operates to count, choose, and initiate X-inactivation has led to a relative neglect of other topics such as that concerning the re-equilibration of levels of expression between the single Xa and autosome pairs. The latter has been suggested to involve the global upregulation of genes on the Xa in both males and females, inducing an increase of 1.4- to 2-fold in expression levels of the X chromosome during the time course of differentiation [185], [186], although a later study involving high-throughput RNA sequencing failed to confirm these observations [187]. Clarification of this important point and a more detailed understanding of the underlying mechanisms are likely to impact largely on current models of both dosage compensation and of the evolution of the sex chromosomes.

The molecular processes responsible for the individualisation of the establishment of a heterochromatin structure on a gene-by-gene basis and the nature of the mechanism(s) rendering “escapees” resistant to global heterochromatinisation or sensitive to reactivation similarly remain, for the most part, unknown. Some of these studies will clearly benefit from the single-cell analyses that will be required to follow in real time the chromatin dynamics occurring during embryogenesis and to capture the putative furtive nuclear interactions and changes in large-scale chromatin organisation that are likely to be part and parcel of the initiation of X-inactivation. Clearly, integrating chromosome-wide and *Xic* nuclear dynamics to transcriptional regulation is but one step in this process. The development of *in vivo* systems allowing the specific perturbation of some of these features/mechanisms during early embryogenesis will, almost certainly, be critical to a complete understanding of how a fully stable Xi is established and how Xi and Xa epigenetic features are transmitted during the formation of mosaic cell populations making up the pre-implantation embryo.
